# Trends in the use of antipsychotics in the Israeli inpatient population, 2004–2013

**DOI:** 10.1186/s13584-016-0074-7

**Published:** 2016-06-15

**Authors:** Alexander M. Ponizovsky, Eli Marom, Michal Ben-Laish, Igor Barash, Abraham Weizman, Eyal Schwartzberg

**Affiliations:** Mental Health Services, Ministry of Health, Jerusalem, Israel; Pharmaceutical Administration, Ministry of Health, Jerusalem, Israel; Research Unit, Geha Mental Health Center and Felsenstein Medical Research Center, Sackler Faculty of Medicine, Tel Aviv University, Tel Aviv, Israel; 35/1 Ha-Kinor St., Ma’ale Adumim, 9837125 Israel

**Keywords:** Atypical antipsychotics, Typical antipsychotics, Sertindole, Ziprasidone, Clozapine, Olanzapine, Quetiapine, Amisulpride, Risperidone, Aripiprazole, Paliperidone, Iloperidone, Pharmacoepidemiology

## Abstract

**Background:**

Although serious mental illneses are treated with both typical and atypical antipsychotic grugs, trends in their use in psychiatric inpatient population in Israel are unrecognized. The aim of this study was to detect trends in the use of typical and atypical antipsychotic drugs in the Israeli inpatient psychiatric population throughout the last decade.

**Methods:**

Data regarding allocation of typical and atypical antipsychotics, over the period 2004 to 2013, were extracted from the electronic records of SAREL, Israel’s largest private supplier of drugs to healthcare and medical facilities. The data were converted to defined daily doses (DDD) per 1000 inpatients per day.

**Results:**

Usage of the ten atypical antipsychotic agents allocated through Israel’s national health care system increased by 73 %, from 128.09 DDD/1000 inpatients/day in 2004 to 221.69 DDD/1000 inpatients/day in 2013. This rise from 2004 to 2013 was largely due to a 1.6-fold increase in the administration of olanzapine (48.31 to 79.57 DDD/1000 inpatients/day), a 4.4-fold increase of quetiapine (9.74 to 43.04 DDD/1000 inpatients/day) and 3.7-fold increase of amisulpride (5.54 to 20.38 DDD/1000 inpatients/day). At the same period, the total utilization of 12 main typical antipsychotics decreased by 15.5 %, from 148.67 DDD/1000 inpatients/day in 2004 to 125.57 DDD/1000 inpatients/day in 2013. Over the entire period, total DDDs of both classes of antipsychotics (typical and atypical) increased by 38 %.

**Conclusions:**

Similar to trends in the treatment of psychiatric outpatients in other countries, there was a substantial increase in the administration of atypical antipsychotic drugs to the Israeli psychiatric inpatient population across the study period. A decrease in the use of typical antipsychotics (substitution), polypharmacy, administration for more indications (supplementation) and the use of larger doses of antipsychotics may account, in part, for this increase. The findings have implications for mental health policy in the context of the Mental Health Care System Reform. Systematic studies on appropriate dosing of antipsychotics and augmentation strategies are warranted.

## Background

Serious mental illnesses (SMI), such as schizophrenia, schizoaffective and bipolar disorders, are chronic medical disorders with unknown etiology, complex pathophysiology and polymorphic symptomatology. Mental, emotional and behavioral manifestations of the disorders result in serious functional impairment, substantially interfering with or limiting one or more major life activities and in turn decreasing quality of life of the patients. Until the mid-1990s only conventional antipsychotic drugs (typicals or first-generation antipsychotics) were used to treat SMI. Atypical antipsychotics (atypicals or second-generation antipsychotics) are currently the first-line treatments for schizophrenia [1, 2] and the recommended maintenance option for bipolar disorder [3, 4]. Both typical and atypical antipsychotic agents block brain dopamine receptors and have comparable efficacy, but atypicals have a safer profile of neurological side-effects: they are less likely to cause extrapyramidal symptoms and tardive dyskinesia [5]. However, atypicals may cause serious metabolic side-effects such as significant weight gain, dyslipidemia and sometimes diabetes mellitus [6].

Since their introduction in the mid-1990s, there has been a proliferation of atypical antipsychotics and there are currently at least 15 atypicals available in psychiatric practice in developed countries [7, 8]. According to the ATC classification index [9], atypicals belong to four chemical groups: 1) indole derivatives (N05AE): sertindole and ziprasidone; 2) diazepines, oxazepines, and thiazepines (N05AH): clozapine, olanzapine, and quetiapine; 3) benzamides (N05AL): amisulpride, and 4) other antipsychotics (N05AX): aripiprazole, iloperidone, paliperidone and risperidone. The introduction of each new atypical antipsychotic agent was accompanied by intense debates among clinicians and researchers regarding the efficacy, side-effect profiles and cost efficiency of the various new drugs [7]. Head-to-head comparisons of atypical antipsychotics lead to the conclusion that atypicals are not a homogenous class [7, 8]. Prescription patterns and, consequently, administration rates of these medications are most likely related to differences in the properties of the atypicals. Clinicians’ prescribing preferences also take into account cost, healthcare policies, marketing, public perceptions, etc.

Over the past decade, several pharmacoepidemiological studies exploring trends in prescription and administration of psychotropic drugs (including atypical antipsychotics) were conducted in different countries [10–13]. Trends were reported for special (e.g., children) and non-institutionalized (ambulatory) populations and were explained either by escalating off-label use or regulatory approval for indications other than schizophrenia and related psychoses [14], such as bipolar mania and depression [15], control of aggression, ADHD and conduct disorders in children [16] and behavioral problems in elderly patients with dementia [17]. In addition, there are a few studies that assessed antipsychotic drug use in inpatient settings [18, 19]. Findings revealed that although the use of atypical antipsychotics dominated inpatient practice, total antipsychotic dosing had not increased from 1998 to 2002 [18]. In ambulatory settings, from 2004 to 2009 total antipsychotic doses increased by 97 %, with better clinical improvement and without apparent increase in major side-effects [19]. Unfortunately, an analogous investigation was not carried out in Israel. Our choice of the targeted population was based on both the availability and reliability of the data on the use of atypicals by psychiatric inpatients and the availability of reliable statistics of psychiatric admissions over the study period.

The psychiatric hospitalization system in Israel includes ten psychiatric hospitals and twelve psychiatric wards in general hospitals. These services provide inpatient care or day hospitalization according to the severity of the patient’s condition. Despite major reform in the patterns of mental health care which resulted in a massive reduction in the number of inpatient beds - from 0.79/1000 population in 2004 to 0.42/1000 population in 2013, the numbers of persons hospitalized yearly over this period declined by only 2.9 % from 18,286 in 2004 to 17,736 in 2013 [20]. Health insurance in Israel is mandatory and is provided by major health maintenance organizations. The drugs covered by the mandatory insurance are included in the National List of Health Services (NLHS). All antipsychotic agents recommended as first-line treatment (except for sertindole) for SMI, are included in the health service list of approved medications. Findings from this study could have important implications for mental health policymakers and cross-country comparisons.

The primary aim of this study was to detect trends in the use of typical antipsychotics (perphenazine, thioridazine, clotiapine levomepromazine, fluphenazine, propericiazine, chlorpromazine, haloperidol, flupentixol, zuclopenthixol, pimozide, and sulpiride) and atypical antipsychotics (sertindole, ziprasidone, clozapine, olanzapine, quetiapine, amisulpride, risperidone, aripiprazole, paliperidone and iloperidone) in the Israeli inpatient psychiatric population throughout the last decade. The second aim was to assess the possible contributors to the trends in the use of antipsychotics. Namely, if there is an increase in use of atypicals, does this occur entirely at the expense of typicals (substitution), or does the introduction of atypicals actually expand the total use of antipsychotics (supplementation)? The latter could occur if psychiatrists are willing to use atypicals for patients or indications where they were reluctant to use typicals.

## Methods

**Fig. 1:**
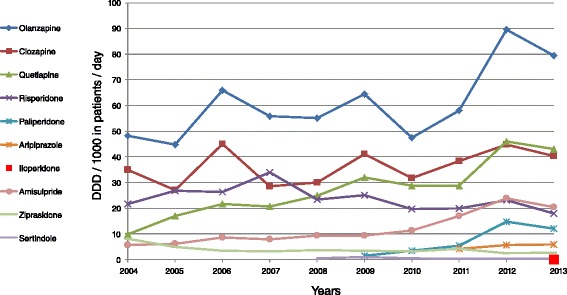
Consumption trends for ten atypical antipsychotics covered by Israel’s national health care system, 2004 – 2013

Drug utilization data were derived from the database maintained by SAREL which is the largest private supplier of drugs to health maintenance organizations and medical facilities in Israel. The company is the only provider of all atypical antipsychotic formulations (allocated by the National Health Insurance system) to all psychiatric hospitals and psychiatric departments in general hospitals in Israel. Data on the total annual administration of the medications (amisulpride, clozapine, olanzapine, quetiapine, risperidone, and ziprasidone) were evaluated for the years 2004–2013. In addition, we analyzed consumption of four other atypicals – sertindole, paliperidone, aripiprazole, and iloperidone that have been marketed in Israel since 2008, 2009, 2011 and 2013, respectively. In parallel to the analysis of the trends in total administration of atypical antipsychotics, we also analyzed the changes in typical antipsychotics over the same period. Dosages for all drugs were converted to defined daily doses (DDD) per 1000 psychiatric inpatients per day, which is the average maintenance dosage as defined by the World Health Organization Collaborating Center for Drug Statistics [21]. DDDs are based on the ATC classification index. [9] To calculate administration rates, we used the formula “number of DDD per 1000 psychiatric inpatients per day = number of packages dispensed × number of doses per package × number of mg per dose × 1000 psychiatric inpatients/DDD in mg x number of psychiatric patients in Israel for the year × 365 days”. Data regarding the total population of psychiatric patients (both inpatients and those in day hospitalization) for each year studied were obtained from the corresponding administrative databases maintained by the Department of Information and Evaluation in the Ministry of Health [20, 22]. The retrospective study did not require Institutional Review Board approval because of the anonymous nature of the data used (without patient ID codes) (Table 1 and Fig. 1).

**Table 1:** Consumption in defined daily dose per 1000 psychiatric inpatients per day of ten atypical antipsychotics covered by Israel’s national health care system, 2004 - 2013

	2004	2005	2006	2007	2008	2009	2010	2011	2012	2013
Olanzapine	48.31	44.69	65.79	55.85	55.14	64.48	47.39	58.04	89.50	79.57
Clozapine	34.87	27.03	45.02	28.44	30.08	41.07	31.59	38.40	44.85	40.22
Quetiapine	9.74	17.01	21.60	20.73	24.90	32.04	28.63	28.71	45.97	43.04
Risperidone	21.61	26.86	26.37	33.99	23.35	25.16	19.52	19.86	23.02	17.79
Paliperidone						1.34	3.42	5.26	14.79	11.98
Aripiprazole								4.04	5.53	5.90
Iloperidone										0.03
Amisulpride	5.54	6.03	8.57	7.71	9.41	9.33	11.29	16.92	23.92	20.38
Ziprasidone	8.02	4.77	3.42	3.18	3.53	3.28	3.24	4.20	2.46	2.69
Sertindole					0.36	1.01	0.35	0.11	0.24	0.11
Total	128.1	126.4	170.8	149.9	146.8	177.7	145.4	175.5	250.3	221.7

The blanks correspond to the periods in which the drugs were not marketed

## Results

**Fig. 2:**
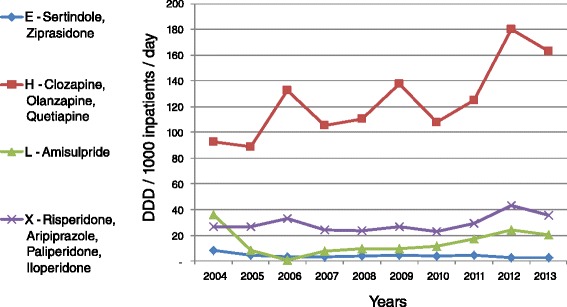
Consumption trends in the ATC atypical antipsychotic drug groups (DDD)

Over the study period, the total annual administration of atypical antipsychotics allocated by Israel’s national health care system increased by 73 %, from 128.09 in 2004 to 221.69 DDD/1000 inpatients/day in 2013. Table 1 and Fig. 1 show that the administration of olanzapine increased by a factor of 1.6, from 48.31 in 2004 to 79.57 DDD/1000 inpatients/day in 2013. The corresponding figures for quetiapine demonstrated a 4.4-fold increase, from 9.74 DDD/1000 in 2004 to 43.04 DDD/1000 inpatients/day in 2013 and for amisulpride a 3.7-fold increase, from 5.54 in 2004 to 20.38 DDD/1000 inpatients/day in 2013. During the same period, clozapine administration increased by 15 %, whereas risperidone administration decreased by 18 %. There was also a 3-fold reduction in ziprasidone administration. For more recently marketed drugs, there was nearly a 9-fold rise in paliperidone administration, from 1.34 in 2009 to 11.98 DDD/1000 inpatients/day in 2013 and a 1.5-fold rise in aripiprazole utilization, from 4.04 in 2011 to 5.90 DDD/1000 inpatients/day in 2013 (Fig. 2).

**Fig. 3:**
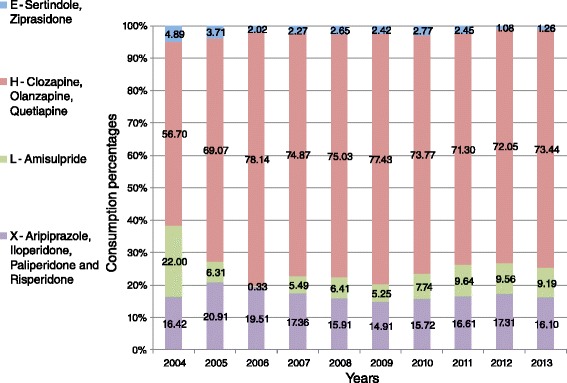
Relative consumption trends across the ATC atypical antipsychotic drug groups

As can be seen in Fig. 2, out of four ATC antipsychotic groups (N05AE; N05AH; N05AL, and N05AX), drug administration, as measured by DDD per 1000 inpatients per day, rose substantially during the study period only in the N05AH group, while in the N05AX group the administration rate increased slightly and there was even some decline in the remaining groups (Fig. 3).

Figure 3 depicts change in proportions of the ATC groups of atypical antipsychotics consumed in Israel over the study period. As can be seen, there is a substantial trend towards increase in the proportions of atypical preparations from the N05AH group including clozapine, olanzapine and quetiapine (from 56.7 % in 2004 to 73.4 % in 2013) alongside reduction in the proportions of atypicals from the N05AE group including sertindole and ziprasidone (4.9 % and 1.3 %, respectively), and the N05AL group including amisulpride (22 % and 9.2 %, respectively). The proportions of atypicals from the N05AX group (risperidone, aripiprazole, paliperidone and iloperidone) remained unchanged (around 16 %) during the study period (Tables 2 and 3).

**Table 2:** Consumption of typical antipsychotics covered by Israel’s national health care system, 2004 – 2013 (defined daily dose per 1000 psychiatric inpatients per day)

	2004	2005	2006	2007	2008	2009	2010	2011	2012	2013
Perphenazine	1.39	1.55	2.04	2.23	2.39	2.38	1.90	2.57	2.64	2.55
Thioridazine	0.42	0.44	0.27	0.32	0.32	0.31	0.28	0.16	0.15	0.18
Clothiapine	6.37	5.27	8.37	5.83	6.59	5.58	6.66	6.64	5.37	4.63
Levomepromazine	0.77	0.95	0.84	0.7	1.19	0.61	0.82	0.77	0.79	1.05
Fluphenazine	70.25	61.17	58.92	57.69	56.86	58.00	54.74	65.53	56.78	57.56
Propericiazine(Periciazine)	0.12	0.14	0.12	0.18	0.22	0.16	0.15	0.13	0.18	0.17
Chlorpromazine	4.00	4.41	3.90	3.31	4.02	3.65	3.69	3.40	3.05	1.52
Haloperidol	42.37	42.31	37.64	45.34	45.75	47.32	45.83	49.04	40.63	38.95
Flupentixol	0.45	0.32	0.25	0.26	0.28	0.18	1.76	0.29	0.11	0.15
Zuclopenthixol	21.05	14.26	16.26	18.79	18.83	19.09	17.59	19.21	17.94	17.58
Pimozide	0.03	0.01	0.07	0.15	0.13	0.12	0.12	0.12	0.01	0.24
Sulpiride	1.40	1.56	2.59	1.58	1.53	1.59	1.10	1.03	1.16	0.99
Total	148.62	132.39	131.27	136.38	138.11	138.99	134.64	148.89	128.81	125.57

**Table 3:** The relationship (%) between DDD of typical and atypical antipsychotics covered by Israel’s national health care system, 2004 – 2013 (defined daily dose per 1000 psychiatric inpatients per day)

	2004	2005	2006	2007	2008	2009	2010	2011	2012	2013
Typicals DDD total	148.62	132.39	131.27	136.38	138.11	138.99	134.64	148.89	128.81	125.57
Percent	53.7	51.2	43.5	47.6	48.5	43.9	48.1	45.9	34.0	36.2
Atypicals DDD total	128.1	126.4	170.8	149.9	146.8	177.7	145.4	175.5	250.3	221.7
Percent	46.3	48.8	56.5	52.4	51.5	56.1	51.9	54.1	66.0	63.8
All	276.72	258.79	302.07	286.28	284.91	316.69	280.04	324.39	379.11	347.27

We assessed the use of atypical antipsychotics against the use of typical antipsychotics at the same time period. As can be seen in Table 2, the total utilization of 12 main typicals (perphenazine, thioridazine, clotiapine levomepromazine, fluphenazine, propericiazine, chlorpromazine, haloperidol, flupentixol, zuclopenthixol, pimozide, and sulpiride) decreased by 15.5 %, from 148.67 DDD/1000 inpatients/day in 2004 to 125.57 DDD/1000 inpatients/day in 2013. The corresponding figures for individual typicals were for Flupentixol 67 %, Chlorpromazine 62 %, Sulpiride 29 %, Zuclopenthixol 16.5 %, and Haloperidol 8 %. Only Pimozide’s use rose by 8 times at that period, from 0.03 DDD/1000 inpatients/day in 2004 to 0.24 DDD/1000 inpatients/day in 2013. However, its portion of total typical antipsychotic consumption was small. Table 3 shows that for the study period the ratio of atypical to typical antipsychotics usage (expressed in percentages) has changed significantly in favor of atypical medications, from 46.3/53.7 % in 2004 to 63.8/36.2 % in 2013.

Regarding the substitution/supplementation question, it was found that over the entire period, total DDDs of both classes of antipsychotics (typical and atypical) increased by 38 %. This shows that in addition to the substitution that is already noted (i.e., the decline in typicals’ share), there was also a supplementation which expanded overall use of antipsychotics.

## Discussion

We investigated the trends in the administration of both typical and atypical antipsychotic drugs in the inpatient population in Israel over the last decade. Results show a substantial increase in the total administration of atypicals, with olanzapine, quetiapine and amisulpride and, recently, aripiprazole and paliperidone accounting for the increase. The administration of older atypicals rose only slightly (e.g., clozapine) or was even somewhat reduced (e.g., risperidone). The growth of atypicals represents both substitution and supplementation, since atypicals’ share increased but so did the overall use of antipsychotics (increased by 38 %). It appears that there was not only substitution of typicals by atypicals, but also use of atypicals for patients or indications in which psychiatrists were reluctant to use typicals.

Similar trends towards increase in the utilization of atypicals have been reported in other countries [12]. For instance, there was 217 % increase between 2000 and 2011 in Australia; [13] the proportion of prescriptions for atypicals rose from 7 % to 96 % between 1996 and 2001 in the United States [16] and from 0 % to 78 % between 1999 and 2005 in Spain [10]. However, unlike our study, the trends were reported for special (e.g., children) and non-institutionalized (ambulatory) populations. The rise in atypicals can be explained by both patterns (substitution and supplementation) [10, 12, 16]. The pattern of supplementation was reflected by either escalating off-label use or regulatory approval for indications other than schizophrenia and related psychoses [14], such as bipolar mania and depression [15], for the control of aggression, ADHD and conduct disorder in children [16] and behavioral problems in elderly patients with dementia [17]. The trends for the increased administration of atypicals to the inpatient population in our study may be explained by some of the above factors, although we focused only on hospitalized patients rather than outpatients.

Comparative efficacy and tolerability of each individual drug should be considered when explaining the more extensive use of certain atypicals. In this respect, a recent multiple-treatment meta-analysis comparing RCTs of 15 antipsychotics [8] provides evidence-based hierarchies for the drugs. According to that analysis, clozapine is significantly more effective than all the other antipsychotics, followed by amisulpride, olanzapine and paliperidone. Clozapine was found to produce fewer extrapyramidal side-effects than all other drugs followed by sertindole, olanzapine and quetiapine, whereas risperidone and paliperidone were among the less tolerated drugs. Hence, the higher administration rates for olanzapine, quetiapine and clozapine in our study could be explained by better efficacy and tolerability. However, olanzapine and clozapine induce significantly more weight gain than most other antipsychotics, and iloperidone, sertindole, quetiapine, risperidone and paliperidone are associated with significantly more weight gain than aripiprazole, amisulpride and ziprazidone. In addition, there are mixed results for the sedative effect that was found to be the highest for clozapine, but low for both amisulpride and paliperidone. Therefore, all-cause discontinuation, which encompasses both efficacy and tolerability, is a more suitable measure of drug acceptability and utility. From this perspective, the significantly lower rates of all-cause discontinuation for olanzapine, quetiapine and clozapine compared to other atypicals [8] could account for their increasing consumption. This explanation is supported by RCTs demonstrating that patients with schizophrenia discontinue antipsychotics mainly due to inefficacy rather than side effects, i.e., patients prioritize efficacy over tolerability [23].

Apart from the above explanations, our findings could be accounted for by the fact that clinicians tend to increase dosing of medications in an attempt to achieve a better response. Prescription of high doses of antipsychotics is common in hospitalized patients, despite the lack of evidence for such an approach [24]. For example, olanzapine and quetiapine are well tolerated at high doses, and these doses may also produce greater improvement [25, 26], whereas increasing the dose of risperidone beyond the optimal recommended dose range (4–8 mg/day) does not improve effectiveness, but exposes the patients to higher rates of side effects [27]. It is possible that our observation of increased administration trends for olanzapine and quetiapine and decreased utilization of risperidone reflect the changes in clinical practice with regard to antipsychotic dosages owing to clinicians’ greater awareness of the balance between clinical benefit and emergent side effects [28].

Another possible explanation for the revealed trends lays in the popular practice among clinicians of administering more than one atypical to enhance treatment effectiveness (polypharmacy). The practice of polypharmacy in the treatment of psychoses is common across countries and clinical settings: [29], 28 % to 57 % of people with schizophrenia reported using more than one antipsychotic during a one-year follow-up [30, 31]. More specifically, Novick and colleagues [32] reported that only 66.8 % of patients treated with olanzapine, 52.6 % treated with amisulpride and 43.4 % treated with quetiapine maintained their baseline monotherapy over 12 months. Thus, polypharmacy of large doses of several antipsychotics may be partly responsible for the increased utilization trends in Israel, at least for antipsychotics. Hence, systematic studies on appropriate dosing of antipsychotics in conjunction with augmentation strategies are warranted.

Health policy-related factors may also explain the observed trends. As mentioned, all atypical antipsychotic agents, except sertindole, are included in the Israeli list of approved medications that are provided at no cost to patients by the mandatory health insurance funds. The inclusion of any pharmaceutical product in the drug list is accompanied by approved indications and limitations detailed in the National Health Insurance Law. Unfortunately, the process of approval of the atypical antipsychotics was made mainly at a bureaucratic level and without the input of an expert committee and thus was not clinical in nature. From 2004 throughout 2013, the clinical indications for dispensation of atypical antipsychotics were extended by the “health basket” committee in the Ministry of Health. Thus, atypical antipsychotics were granted priority over other treatment options (typical antipsychotics) encouraging the use of atypicals, and consequently, increasing their rates of administration. This was true for olanzapine, quetiapine and amisulpride which were approved in 2006 as first-line treatment for both schizophrenia and bipolar disorder. Similarly, the administration of paliperidone and aripiprazole rose following their inclusion in the approved drug list in 2009 and 2010, respectively.

Atypical (second-generation) antipsychotics were introduced in Israel in 2000 and their cost was substantially higher than that of first-generation antipsychotics. Restricted use of atypicals reflected the significant budget constraints of psychiatric hospitals. Despite the burden, clinical reality required the use of atypicals to attenuate the exposure to serious adverse effects (mainly extrapyramidal) of the first-generation antipsychotics. However, it is of note that treatment with atypical antipsychotics is associated with obesity, diabetes and metabolic syndrome. The medical care system’s cost of reducing EPS by going over to second generation atypical antipsychotics in terms of metabolic abnormalities should be investigated in future studies. It is possible that the results of such studies could reveal a historic mistake made in psychiatry.

It should be noted that the initial high costs of all new atypical antipsychotics substantially decreased five years following approval, which coincided with the introduction of their generic counterparts. Thus, the increase in administration of atypicals can be attributed in part to the registration of less expensive generic drugs in Israel. The introduction of generic olanzapine in 2011 most likely accounted for the sharp increase in its administration owing to the reduced cost.

The trends in the use of atypical antipsychotics reported in this study provide useful information for policy development for the treatment of patients with SMI, patients resistant to conventional treatment or those experiencing serious adverse effects.

### Policy implications for the near future

The National Health Insurance Law, introduced in Israel in 1995, exposed a gap between provision of physical and mental health services. Article 2 of the Law assigned responsibility to the Health Maintenance Organizations (HMOs) for physical health services. However, according to Article 3, the State remained responsible for mental health services. The Mental Health Service System Reform [33] declared that the State would finance psychiatric hospitals until July 2015, after which financial responsibility would be transferred to the HMOs. Psychiatric hospital budgets include salaries for staff (based on collective agreements) and for all hospital operations, including medications.

Given the above considerations, the Israeli Ministry of Health allocated an earmarked 10 million NIS annual budget for atypical antipsychotic agents. Over the last decade, the number of new atypical antipsychotics increased and their costs decreased following the introduction of generic forms of some of the atypicals. These parallel processes allowed the psychiatric hospitals to extend the use antipsychotics and remain within the constraints of the existing budget.

The Mental Health Reform implemented fundamental changes in the economic status of state psychiatric hospitals: HMOs now finance all psychiatric hospitalizations, and the former state-owned hospitals no longer receive government funding. Prior to the Mental Health Reform, psychiatric hospitals received an earmarked budget for atypical antipsychotics (approximately 14.5 million NIS total, or 2400 NIS per bed). Following implementation of the reform, this special budget was cancelled and the allotment was added to the cost per day of hospitalization. Considering that most of the drug company profits are from medications administered in ambulatory care, negotiations are under way with the pharmaceutical companies to reduce the cost of inpatient medications. Economic limitations and inevitable budget deficits will undoubtedly impact the choice of inpatient antipsychotic treatment.

The use of clozapine will be extended for both acute and chronic resistant patients for two reasons. First, clozapine is the most effective treatment for schizophrenia patients who are resistant to other atypicals, and second, its generic form is relatively inexpensive. It is therefore expected that the HMO’s will encourage psychiatric hospitals to initiate the treatment with clozapine as early as possible, an advantageous scenario for schizophrenia patients and their families.

Long-acting forms of atypical antipsychotics will be prescribed mainly in community health-care settings as maintenance treatment for schizophrenia and will be prescribed in hospitals just prior to discharge in line with HMO policy.

In sum, the trends detected in this study support several recommendations for policy development regarding the treatment of patients with SMI, patients resistant to conventional treatment or those experiencing serious adverse effects of conventional treatment.

### Study limitations

The calculation of the defined daily dose (DDD) adjusts total consumption of atypical antipsychotic medications by the number of psychiatric inpatients nationwide. Hence, variation in DDDs over time could reflect changes in the volume of inpatients with disorders other than schizophrenia, many of whom are less likely to be treated with atypical antipsychotics. This could account for the large year-to-year swings in DDDs, if the total psychiatric inpatient population varies more over time than the size of the inpatient population with schizophrenia.

In addition, our study included only inpatients in all psychiatric settings (i.e., the most severe cases), hence the obtained results cannot be generalized to outpatients with SMI or the general population. As previously mentioned, this study sample was chosen because of the availability of reliable data on atypical antipsychotic utilization in the inpatient population and the reliable statistics of psychiatric hospitalizations. Future studies targeting outpatient populations are warranted.

Another limitation is the use of an aggregate, non-patient level of data. Due to this limitation the contribution of growth in polypharmacy, increasing doses over time, or inclusion of more patients receiving such treatments could not be assessed. Furthermore, the aggregate data evaluation cannot scrutinize explanations for changes in the prescribing patterns of atypical antipsychotic agents. Some of the factors that drive drug-specific prescribing trends are not observable in aggregate data. As a result, a further study could focus on explanatory factors that can be related to the aggregate data, such as the timing of introduction of various drugs to the market, and the dates that they were first introduced as generics. A timeline with those changes could add considerable value to the study by illustrating which regulatory changes relate to which trends. Unfortunately, we do not have reliable information regarding the timing of drug and generic entry. The use of patient-level data is an interesting topic for future study, in which the number of patients using each medication could be analyzed.

Finally, the time-frame for the trends of recently marketed atypicals (paliperidone, iloperidone, and sertindole) is shorter than for the older atypicals, such as clozapine, olanzapine and risperidone. Further research is needed to explore the consumption trends for the novel atypicals in the long-term.

It appears that the growth in the use of atypicals represents both substitution for typicals as well as a supplementation (expansion of the total use of antipsychotics), namely, use of atypicals for patients or indications where they were reluctant to use typicals. A 10-year retrospective study on the trends in prescribing psychotropic drugs to children and adolescents within an inpatients adolescent psychiatric ward in Israel has demonstrated that typical antipsychotic prescriptions decreased by 35.5 % while the prescriptions of atypical antipsychotics increased by 51.5 % [34]. Similar analysis of the trends in the types of antipsychotic prescribed to schizophrenia patients over a 3-year period has been found in two large mental health catchment areas of Auckland (Australia): with an 18.6 % increase in atypical antipsychotics and a 23.3 % decrease in both intramuscular and oral typical antipsychotics [35].

## Conclusion

The reasons for the increasing administration of atypical antipsychotics, as well as the decrease in the consumption of typicals, in the psychiatric inpatient population over the last decade are not totally clear. Similar trends have also been observed in other countries among psychiatric outpatients. These trends seem to be related to both substitution and supplementation. In the absence of evidence on the nature of the increasing trends in the utilization of atypical antipsychotics among inpatients, the possible contribution of their extended off-label prescription or potential augmentation strategies of combinations of several antipsychotics (polypharmacy) cannot be ruled out. Rigorous detailed research in dosing of antipsychotics and their combined use is needed before firm conclusions for solid policy changes can be drawn.
